# Major Depressive Disorder and Patterns of Antidepressant Prescription in Greece: Α Pharmacoepidemiological Study

**DOI:** 10.1192/j.eurpsy.2026.10426

**Published:** 2026-07-31

**Authors:** R. I. Gournellis, I. Tollos, C. Doukas, M. Hadjulis, I. Michopoulos, P. Mitrou, E. Thireos, K. Mathioudakis, P. Skapinakis, V. Karageorgiou

**Affiliations:** 1 Second Department of Psychiatry; 2 Faculty of Nursing, National and Kapodistrian University of Athens; 3 Hellenic Ministry of Health; 4 National Agency for Quality Assurance in Health S.A; 5 IDIKA SA - e-Government Center for Social Security Services, Athens; 6 Department of Psychiatry, University of Ioannina, Ioannina, Greece

## Abstract

**Introduction:**

Psychopharmacological **e**pidemiological data regarding Major Depressive Disorder (MDD) are based on small-scale representative studies.

**Objectives:**

Our study aims to determine the one-year prevalence of major depressive disorder under antidepressant treatment, identify the severity of MDD, and describe patterns of antidepressant prescription for MDD in Greece.

**Methods:**

The electronic prescription database of the e-Government Centre for Social Security was used to describe the characteristics, the diagnoses and the prescription patterns in patients that received at least two prescriptions of antidepressants, with an ICD-10 code relevant to unipolar depression, during the 1-year period between 1st of February 2019 and 31st January 2020. All the beneficiaries of the National Organization for Health Care Services Provision were covered.

**Results:**

The study population (n=369,860) indicates a 1-year prevalence of antidepressant-prescribed unipolar depression of 3.5%, with clear predominance of females (females: 72.5%), and mean sample age 65.1 years (standard deviation: 15.3). Concerning the severity of the diagnoses, 52.0% of the sample was characterized as “mild-moderate” severity, 6.6% was characterized as “severe” severity, and 56.2% was characterized as “unspecified” severity. 86.2% of the patients received one antidepressant, 12.1% of the patients received two antidepressants, and 1.7% of the patients received at least three antidepressants. The most commonly prescribed category of antidepressants was “SSRIs”, (78.3% ), followed by “SNRIs” (17.1%).

**Conclusions:**

The prevalence of antidepressant-prescribed MDD was estimated at 3.5%. Identifying the prescription patterns of antidepressant treatment for MDD is of paramount importance in formulating policies for a cost-effective and quality-acceptable management of the disorder.

**Disclosure of Interest:**

None Declared

